# Supplementary material for: “It’s my life, not theirs!” Therapeutic itineraries and refugee reflections on referral health care in western Tanzania
Source: PLOS Glob Public Health. 2023 Jun 8;3(6):e0001655. doi: 10.1371/journal.pgph.0001655 (PMC10249889; doi:10.1371/journal.pgph.0001655)
Supplement: Attachment — Submitted filename: Response to Reviewers_7Feb.docx [file pgph.0001655.s001.docx]

Response to Reviewers:

Journal Requirements:

1. Please provide additional details regarding participant consent. In the ethics statement in the Methods and online submission information, please ensure that you have specified what type you obtained (for instance, written or verbal, and if verbal, how it was documented and witnessed). If your study included minors, state whether you obtained consent from parents or guardians. If the need for consent was waived by the ethics committee, please include this information.

Thank you. This has been added to the Methods section that it was verbal consent that was obtained.

2. Please amend your detailed Financial Disclosure statement. This is published with the article. It must therefore be completed in full sentences and contain the exact wording you wish to be published.

Thank you. This has been amended to read as a full sentence.

a. Please clarify all sources of funding (financial or material support) for your study. List the grants (with grant number) or organizations (with url) that supported your study, including funding received from your institution.
   b. State the initials, alongside each funding source, of each author to receive each grant.
   c. State what role the funders took in the study. If the funders had no role in your study, please state: “The funders had no role in study design, data collection and analysis, decision to publish, or preparation of the manuscript.”
   d. If any authors received a salary from any of your funders, please state which authors and which funders.

This information is included in the Declarations section.

3. Please provide separate figure files in .tif or .eps format.

For more information about figure files please see our guidelines:

<https://journals.plos.org/globalpublichealth/s/figures> 
<https://journals.plos.org/globalpublichealth/s/figures#loc-file-requirements>

Thank you. The image is uploaded as a .tif file.

3. We have noticed that you have uploaded Supporting Information files, but you have not included a list of legends. Please add a full list of legends for your Supporting Information files after the references list.

Thank you. The only file is the figure.

5. In the online submission form, you indicated that "Data used and analyzed in the current study are not publicly available due to privacy and personally identifiable health information that could compromise the participants who were interviewed in the study. Data could potentially be de-identified upon reasonable request but data was obtained in Kiswahili language.". All PLOS journals now require all data underlying the findings described in their manuscript to be freely available to other researchers, either 1. In a public repository, 2. Within the manuscript itself, or 3. Uploaded as supplementary information.
This policy applies to all data except where public deposition would breach compliance with the protocol approved by your research ethics board. If your data cannot be made publicly available for ethical or legal reasons (e.g., public availability would compromise patient privacy), please explain your reasons by return email and your exemption request will be escalated to the editor for approval. Your exemption request will be handled independently and will not hold up the peer review process, but will need to be resolved should your manuscript be accepted for publication. One of the Editorial team will then be in touch if there are any issues.

The protocol approved by our institutional IRB does not permit sharing of the confidential information obtained in this study. Further, direct patient names are used throughout the interview process and script. As the author, I am fluent in Kiswahili and English and not all interviews were formally transcribed into English written form. Nevertheless, given this is an already vulnerable population (refugees in a post-socialist state), I humbly request to avoid full sharing or any publication of the data source. It is a fairly small number of individuals interviewed, but the specific health details shared may make someone identifiable. Thank you for your consideration.

Additional Editor Comments (if provided):


[Note: HTML markup is below. Please do not edit.]

Reviewers' comments:

Reviewer's Responses to Questions

**Comments to the Author**

1. Does this manuscript meet PLOS Global Public Health’s [publication criteria](https://nam02.safelinks.protection.outlook.com/?url=https%3A%2F%2Fjournals.plos.org%2Fglobalpublichealth%2Fs%2Fcriteria-for-publication&data=05%7C01%7Czoe%40jhmi.edu%7C17662bf9ae704b1d7c2308db0538dafe%7C9fa4f438b1e6473b803f86f8aedf0dec%7C0%7C0%7C638109513969507978%7CUnknown%7CTWFpbGZsb3d8eyJWIjoiMC4wLjAwMDAiLCJQIjoiV2luMzIiLCJBTiI6Ik1haWwiLCJXVCI6Mn0%3D%7C3000%7C%7C%7C&sdata=5K6mqpBUGTbCecGSVOiUnePuzPAiSTKzCu1PPS82NTI%3D&reserved=0)? Is the manuscript technically sound, and do the data support the conclusions? The manuscript must describe methodologically and ethically rigorous research with conclusions that are appropriately drawn based on the data presented.

Reviewer #1: No

Reviewer #2: Yes

2. Has the statistical analysis been performed appropriately and rigorously?

Reviewer #1: No

Reviewer #2: N/A

3. Have the authors made all data underlying the findings in their manuscript fully available (please refer to the Data Availability Statement at the start of the manuscript PDF file)?

The [PLOS Data policy](https://nam02.safelinks.protection.outlook.com/?url=https%3A%2F%2Fjournals.plos.org%2Fglobalpublichealth%2Fs%2Fdata-availability&data=05%7C01%7Czoe%40jhmi.edu%7C17662bf9ae704b1d7c2308db0538dafe%7C9fa4f438b1e6473b803f86f8aedf0dec%7C0%7C0%7C638109513969507978%7CUnknown%7CTWFpbGZsb3d8eyJWIjoiMC4wLjAwMDAiLCJQIjoiV2luMzIiLCJBTiI6Ik1haWwiLCJXVCI6Mn0%3D%7C3000%7C%7C%7C&sdata=vkCWZ%2FuGr22Da1IVVgWvS3SpkAW0C0JVKkBNJgDZCQA%3D&reserved=0) requires authors to make all data underlying the findings described in their manuscript fully available without restriction, with rare exception. The data should be provided as part of the manuscript or its supporting information, or deposited to a public repository. For example, in addition to summary statistics, the data points behind means, medians and variance measures should be available. If there are restrictions on publicly sharing data—e.g. participant privacy or use of data from a third party—those must be specified.

Reviewer #1: No

Reviewer #2: No

4. Is the manuscript presented in an intelligible fashion and written in standard English?

PLOS Global Public Health does not copyedit accepted manuscripts, so the language in submitted articles must be clear, correct, and unambiguous. Any typographical or grammatical errors should be corrected at revision, so please note any specific errors here.

Reviewer #1: Yes

Reviewer #2: Yes

5. Review Comments to the Author

Please use the space provided to explain your answers to the questions above. You may also include additional comments for the author, including concerns about dual publication, research ethics, or publication ethics. (Please upload your review as an attachment if it exceeds 20,000 characters)

Reviewer #1: An important topic and very relevant study, however, due to the wide differences in country specific policies on refugee freedom of movement, and the impact of these policies on the answers to the research questions, more specific methodological details are required before the manuscript will add to the body of knowledge in this field.

Thank you for your kind and thoughtful review. Please see below for my detailed responses.

Some specific questions are: What were the selection criteria for the participants and what were the demographic specifics of the participants? Why were only two Tanzanians selected if the author wanted to make comparisons with the standard of care for citizens. In fact, the comparison detracts from the study as it is not explored in enough detail. I would remove the comparison and focus on the conditions for refugees and compare in more detail to the policies and lack of adherence to the policies.

Thank you for these questions and points of clarification. To respond, the participants were chosen from a larger registry of patients requiring referral health care. In the refugee camp, each time a referral is made, a formal referral form must be filled out by a physician and then this is subsequently prepared for presentation at the monthly meeting. These referral forms were reviewed and patients selected based on various pathologies, but not all patients selected were available. This has been further reflected in the updated manuscript.

It was quite difficult to physically track any Tanzanian patients regarding referral. This is in large part because the Tanzanian patients who present to the hospital do not necessarily stay in the hospital or camp once a referral is made, as they have freedom of movement and can return to their homes or on to the hospital/health center higher up in the health care hierarchy. Despite multiple attempts, it was also hard to keep track of any way to contact the Tanzanian patients. Your point, then, on removing the comparison is very well-received. This has now been removed. What I have added in its place is a more concrete argument for how policies on freedom of movement (to your introductory comments) are unique to Tanzania, as this comparison, in my opinion, is an important one and addresses your comment that the conditions and refugees are unique and different and the policies on freedom of movement are different. This can be found in the Discussion section.

The Methods section requires greater specific details in order to prevent the self-confessed bias of the author. The data collection methodology and data analysis need more rigorous description (e.g. research assistant’s skills and training for data collection, analysis methods and findings from the memos and summaries, reliability and validity methods used for both data collection and analysis).

Thank you for this comment. I have added some more detail on the methodology and my approach to analysis, from a theoretical and practical standpoint. Respectfully, I do think one strength of the study is approaching referral health care from an anthropological lens, rather than a more quantitative or objective epistemology. What I mean to say is while there is a role for reliability methods in certain types of quantitative and qualitative studies, the goal of this study was to utilize the theoretical frameworks of biopower and therapeutic itineraries and apply them to this particular context of referral health care in western Tanzania. As Trostle has outlined in their text *Epidemiology and* Culture (see Chapter 3), the strength of anthropology is seeking a co-created truth (read: validity), between participant and observer/researcher by utilizing other methods and triangulation of data, such as archival/document review and participant observation, in addition to interviews. I have added more background and justification of this in the methods section.

To be more specific, I am fluent in Kiswahili and English, and thus as the sole person analyzing the data, not all data was necessarily transcribed or translated into English. I have reflected this more accurately now in the updated text, as I believe the previous version had stated/implied that all interviews were formally translated/transcribed. Please forgive this oversight/mistake and please let me know if this poses some issue to the reviewers or editors. I have also added more detail on the research assistant’s background and training. Thank you for these important points.

Since the findings of this study can only be applied in specific refugee contexts, it becomes very important to strengthen the conclusions derived from the study. Therefore, a very clear link between each research question and the answers found in the study and then most importantly, the policy and practice implications of the findings would be necessary if this study is to address the gap in the field/topic. A Policy brief to the Tanzanian government should be the obvious outcome of this study and therefore evidence of more rigorous qualitative scientific methodology needs to be evident in this manuscript.

Thank you for this comment. I am in complete agreement. I have attempted to allude to these points on what might be found in a brief to the government. On a practical micro level, this could look like better communication and tracking systems. On a macro level, revisiting restriction of movement or seeing how other countries manage chronic or NCDs could be useful. I am attempting in the article to provide and use language that would not potentially put at a disadvantage some of our current efforts to carry out research and impede any of our collaborators who facilitated our research, as the Tanzanian government has to grant a permit for us to enter the refugee camp. See Conclusion section.

Reviewer #2: This is an original idea on an area of which we have little knowledge or understanding. How refugees experience referral and transfer to care is of meaningful concern for the efforts, convolutions and potential delays that can impact on health outcomes. The manuscript is well written and the narratives are compelling. The opening section is particularly well-written and captivating.

Thank you for these kind comments.

A few small comments:

1 - BOX-1 contains what appears to be detailed medical notes of a particular patient. The author indicates that her name has been changed, however, the details appear to include address and location of her camp, as well as detailed health information. I am not sure this is consistent with confidentiality requirements, especially since the the study data is not made available to protect patient identities. If, however, the information is invented for the purposes of the study, I suggest cutting most of it, since addresses and patient numbers are not relevant to the story being told.

Thank you. Yes, all of the detail in that that would be identifying (name, address, etc.) are fabricated. For example, there is no zone 19 in the camp. I have deleted most of the fabricated but potentially identifying information and also noted in the box that the name is changed for anonymity reasons. Thank you.


2 - A bit more detail about the methods and design of study would be welcome. Not much is needed for space, but it is not clear if this was a partly ethnographic study, or if it is a grounded theory approach to a qualitative study etc.

Thank you. I have include a few more details per the comments for Reviewer 1 above. It is not formally a grounded theory study, as I purposefully approached the analysis with a lens towards biopower and therapeutic itineraries. These theoretical frameworks are outlined in detail in the Methods section, although if the reviewer/editors feel that more detail is needed, I am happy to provide. I would argue this is ethnography given extended (although, non-contiguous) time in the camp utilizing methods of interviews, participant observation and document review, as well as constantly being in touch with camp staff even when not physically present. I have added some of this language to the methods.

3 - Although the author indicates that care for non-refugee patients would be very different, it would be helpful to understand if the referral delays and problems are exclusively the experience of refugees or if community members attending the same hospitals have similar problems? Are the issues unique to refugee experience? Are local patients experiencing different issues, such as finding the funds to travel too a hospital in another district?

Thank you for this comment. Per the Reviewer 1 comments, I have removed some of the comparison to Tanzanian citizens, but I have alluded to some of this now in the discussion section. Tanzanian patients may face significant delays in care due to cost and while politically they have freedom of movement, they would have to cover the cost of care, apart from if/when they are treated in the refugee camp setting (a reason why many of them go to the camp for treatment).

4 - I know that in other communities, e.g. Jordan, there is emphasis on parody so that refugees are never seen to receive more than local communities. It would be interesting to draw some high level comparison with other settings if possible.

Thank you. In our other work, we have suggested that around 25% of all surgeries going on in the camp are for Tanzanian patients. This was about 10,000 major operations in year 2000 to 2020. I added this citation to the paper now and also have added a small comparison to the Jordanian context regarding complex care, such as cancer.

Overall this is a welcome contribution to literature on refugee health and continuity of care for refugee populations. I strongly support publication of this paper and look forward to being able to share it with others.

Thank you kindly for your comments to improve the article.
